# Challenges in the profitability of small-scale broiler farming by avoiding injudicious use of drugs and additives

**DOI:** 10.1016/j.heliyon.2024.e25001

**Published:** 2024-01-23

**Authors:** Shubash Chandra Das, Mosammad Zubiatin Tasmin, Afifa Afrin, Tanvir Ahmed, Ankon Lahiry, Shahina Rahman

**Affiliations:** Department of Poultry Science, Bangladesh Agricultural University, Mymensingh, 2202, Bangladesh

## Abstract

The aim of the current study was to determine the present scenario of injudicious use of drugs and additives in small-scale broiler operations and whether broilers can be produced successfully and profitably without the injudicious use of drugs and additives. First, a survey was undertaken in relation to farmers' basic information and general management methods in commercial broilers, with special attention given to the usage of medications and additives in drinking water. Second, based on the survey data, an experimental trial was carried out to compare the growth performance and economic profitability of rearing broilers with and without the use of said drugs and additives. A total of 540 broiler DOCs were allotted into three treatments: T_1_ = self-formulated feed (SFF) with judicious use of drugs and additives; T_2_ = commercial feed with judicious use of drugs and additives (JUDA) and T_3_ = commercial feed with injudicious use of drugs and additives (InJUDA), with six replications (30 birds/replication) in each. The results showed that the farmers used a variety of drugs and additives in 35 days of broiler rearing; however, the farmers usually did not consult with veterinary practitioners, instead relying on and being instructed by local dealers and medicine company representatives. Although the medications and additives account for almost 6–8% of total production costs, the experimental trial clearly demonstrated that the broilers kept with either JUDA or InJUDA showed statistically (p < 0.05) similar BW (2181.93 g & 2222.53 g/bird), BWG (2110.0 g & 2129.91 g/bird), and FCR (1.62 & 1.57, respectively), whereas broilers in the SFF group showed significantly lower growth performances (BW = 1799.31 g/bird, BWG = 1746.19 g/bird, and FCR = 1.93, respectively). The net profit per kg bird in the JUDA group was substantially (p < 0.05) greater (BDT- 27.34/-), followed by the SFF group (BDT- 25.56/) and the InJUDA group (BDT- 24.49/-). Taken together, these findings suggest that profitable broiler farming is possible without the injudicious use of drugs and additives.

## 1 Introduction

As an animal protein source, broiler meat alone contributes approximately 40 % of total meat consumption, which is ensured by producing approximately 17.5–20.0 millions of broiler DOCs per week [1]. Small- and medium-scale farms continue to dominate commercial broiler production in Bangladesh, accounting for approximately 81 % of overall production [2]. However, it is a harsh reality that small-scale farmers who raise broilers in vulnerable situations are always denied access to proper guidance and/or a package of suggestions from the concerned authority, experts, or other relevant personnel; rather, they are tempted and encouraged by dealers, medical representatives, and other local agents to use drugs and additives indiscriminately in broiler operations [3,4]. Farmers are completely reliant on local agents and/or dealers due to their inability to purchase DOCs from hatcheries and feed from feed mills [5]. As a result, dealers or other local agents take full advantage of farmers' dependence and obtrude them through primary or secondary means to meet their selling aim by offering drugs (antibiotics), additives, and other inputs injudiciously without considering the needs of birds. Most of the small and medium-scale farmers used antibiotics for therapeutic purposes without consulting a veterinarian; rather, they used the drugs and additives sparingly for both therapeutic and subtherapeutic (growth promoter) purposes, following the advice of dealers, local agents, and other farmers [3,6]. The drugs and additives used are rationally thought to be unnecessary in most instances, and they have not only negative effects on consumer health but also negative consequences on broiler meat quality and farmers' profitability. Such indiscriminate use of antibiotics or additives in broiler production is causing bacterial resistance to antibiotics and is linked to major worldwide health concerns. A published report also mentioned that the issue of safe chicken meat and egg production in Bangladesh should be more sufficiently addressed [7]. Scientists predict that the spread of highly pathogenic drug-resistant bacteria will kill over 10 million people by 2050, with the majority of these deaths occurring in Africa and Asia [8,9]. Keeping these facts in mind, the European Commission (EC) banned the marketing and use of antibiotics as growth promoters in livestock and poultry in 2006 (EC Regulation No 1831/2003), while Bangladesh's Animal Feed Act banned the use of antibiotics in feed in 2010 [10]. Since most nations banned the use of antibiotics as growth promoters in the previous decade, researchers have focused on identifying alternatives such as garlic, phytonutrients, fennel, garlic, oregano, mint, and rosemary that do not have an inimical effect on poultry production [11,12]. However, some farmers in Bangladesh continue to use antibiotics and additives due to high inciting intensity by dealers and other middlemen. To the best of our knowledge, no approaches have yet been taken to determine the actual scenario of small- and medium-scale broiler farming and how much profit is lost owing to the injudicious use of drugs and additives. It is also uncertain how much farmers are influenced by dealers, local agents, or practitioners to use such medications and additives. As a result, a survey was carried out to assess the existing scenario of drug and additive use, and a study was conducted based on this survey to assess whether the irrational use of drugs and additives yield satisfactory growth of broilers in small-scale farm operations.

## 2 Materials and methods

### 2.1 Phase-1: survey on the present scenario of using drugs and additives in small-scale broiler farming

#### 2.1.1 Location of the study area, participants, and sample size

This survey was conducted in an area where small- and medium-scale broiler farming is predominant. Mainly rural and peri-urban areas of Fulbaria, Jamalpur, Trishal, and Bhaluka Upazilas under the districts of Mymensingh and Jamalpur were surveyed. Fulbaria Upazila is approximately 27 km from the metropolis of Mymensingh. It has a total area of 402.41 square kilometers, with 7 unions, 18 mouzas, and 82 villages, with a population of 4,48,462 people (density 1100/km2), and approximately 27 % of them were dependent on livestock farming (Wikipedia). *Jamalpur Sadar Upazila* covers 489.56 square kilometers and has a population density of 1300 people per square kilometer (total of 6,15,072). It contains 8 *Unions*, 173 *Mouza*, and 179 villages, with livestock farming accounting for 34 % of the population (Wikipedia). *Trishal* Upazila, located approximately 21 km from Mymensingh, has a total size of 338.98 square kilometers, with a population of 419,308 people, only 40.2% of whom are literate. The majority of inhabitants in *Trishal* Upazila make their living from poultry and fish farming. *Bhaluka* is 70 km away from Dhaka, with a total size of 444.05 square kilometers and a population of 430,320 people, with a literacy rate of approximately 50 %. These four Upazilas were chosen for data collection because small- and medium-scale broiler farming is concentrated and predominant in these areas. Farmers who commercially reared less than 5000 (small scale) or 10,000 (medium scale) birds were chosen for an in-depth face-to-face interview. We obtained a list of small- and medium-scale poultry farmers from the corresponding *Upazilas* and selected 100 farms (n = 100). Finally, we visited the farms and conducted in-depth interviews with the farmers using a structured questionnaire to obtain the necessary information.

#### 2.1.2 Data collection

As previously stated, a structured questionnaire was employed to collect data. Face-to-face interviews were conducted with 100 respondents who were actively involved in the operation of small and medium-sized broiler farms. The questionnaire was designed to capture the type of information required to achieve the survey objectives. Once the survey goals and associated data demands were determined, an interview schedule was created to record the information needed for analysis. The questionnaire was pretested to judge its suitability before fixing the schedules of respondents. Based on the results of the pretested survey, necessary changes were made by inclusion or exclusion of information from the draft schedule. Then, the draft schedule was improved, rearranged, and finalized in light of practical field experience. Furthermore, attention was given to the general form of the interview schedule to ensure that the question followed a logical and appropriate sequence. Special care was also taken in the wording of questions to ensure that the farmers were not to be unclear at any stage of interviewing. The survey contained both open and closed forms of questions. The farmer's general information, farming status, collection of feeds and DOCs, bird's management procedure, existing live bird marketing, and finally tabulation of the listed antibiotic and nonantibiotic drugs, additives, and other supplements used in broilers were all included in the questionnaire. The data collection was performed in *Bengali*. Audio recordings of interviews were made, and a field note was kept for recording further information. Drugs and additives used by farmers were photographed to double-check with the list.

### 2.2 Phase-2: experimental trial for the evaluation of necessity in the use of drugs and additives

#### 2.2.1 Ethical approval

The Animal Welfare and Experimentation Ethics Committee of Bangladesh Agricultural University, Mymensingh 2202, Bangladesh (Ref. No: AWEEC/BAU/2023 (26)), approved all methodological protocols and experimental design used for the trial.

#### 2.2.2 Bird management and experimental layout

The experiment was conducted at the Bangladesh Agricultural University Poultry Farm, Mymensingh, Bangladesh. A total of 540 DOCs were collected from a reputed commercial hatchery and allocated into three treatment/groups with six replications in each treatment and 30 birds per replication. The treatments were T_1_: self-formulated feed (SFF) with judicious use of drugs and additives [13], T_2_: commercial feed with judicious use (JUDA) of drugs and additives, and T_3_: commercial feed with injudicious use (InJUDA) of drugs and additives. In T_3,_ injudicious use of drugs and additives refers to the list of drugs and additives that are conventionally used by small-scale farmers in their routine farm management practices and that are prescribed and/or compelled to use by the relevant dealers. Before the chicks arrived, a gable-type open-sided chicken house was thoroughly cleaned, washed, disinfected, and dried. The entire room was sterilized with Virkon S (@ 1:100 v/v; DuPontTM Virkon® S, Antec International, USA), a common but frequently used efficient disinfectant purchased from the local market. Clean, dry, and fresh rice husk was employed at a depth of 5 cm as bedding materials. To prevent the accumulation of moisture as well as ammonia and other dangerous gases, litter material was racked at least twice daily. Furthermore, one 200-W bulb and one 100-W bulb were hung in each pen to achieve the appropriate brooding temperature (32–33 °C). Each day, the chicks were exposed to 23 h of light and 1 h of darkness. While brooding was performed, several management approaches such as shutting off one or both lights, adjusting light position (up/down), and curtain withdrawal were employed to maintain appropriate temperature and humidity. The room temperature and humidity were measured using an automated thermohygrometer. On Day 4, the birds received an IB + ND vaccination (CEVAC® BIL, which comprises the Massachusetts B48 strain of infectious bronchitis virus and the Hitchner B1 strain of Newcastle disease virus in live, freeze-dried form), followed by a booster dose on Day 20. Infectious Bursal Disease (IBD) vaccination (GumboMed™ Vet, containing live attenuated Infectious Bursal Disease virus intermediate strain) was also administered on Day 11, with a booster dose administered on Day 18.

#### 2.2.3 Experimental diets, feeding, and watering

**Fig. 1:**
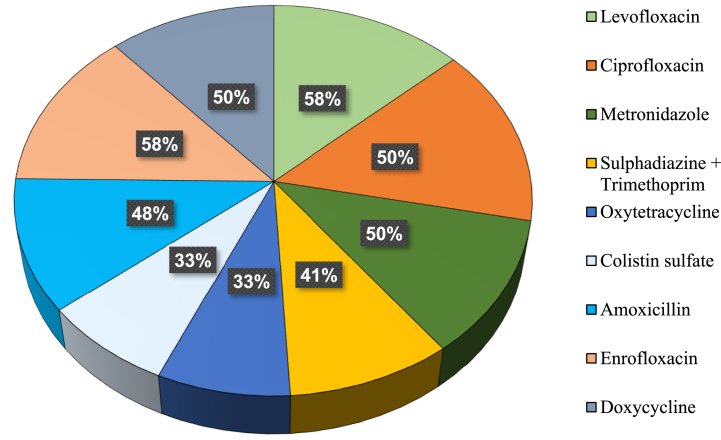
Percentage of antibiotics used by small and medium scale broiler farmers.

For the first treatment group, self-formulated starter and grower diets were made in accordance with the feeding standard of Indian River Meat broilers [13; Table 1]. The starter diet was supplied for the first 14 days, followed by a grower diet for the rest of the rearing period, i.e., 15–35 days. Balanced diets, both starter and grower, were formulated using fresh, good-quality feed ingredients purchased from the local market. In treatment 2, the birds were fed commercial-ready feed with judicious use of drugs and additives (JUDA) as prescribed by a registered veterinary doctor. In treatment 3, on the other hand, experimental birds were fed the same commercial feed but with a list of injudicious use of drugs and additives (InJUDA), as conventionally practiced by small- and medium-scale farmers that were observed in survey areas (Fig. 1). The proximate composition of the self-formulated feed samples was determined using the Bates J. AOAC method [14]. For treatments 2 and 3, birds were fed a broiler prestarter diet for the first 14 days, then a starter diet for up to 21 days, and finally a grower diet for the remaining period (Table 2). Commercial-ready feed was purchased from Nourish Poultry and Hatchery Limited, Bangladesh. The DOCs were transferred, weighed, and randomly distributed in each pen soon after they arrived. The birds were given vitamin C-supplemented glucose saline in their drinking water to provide rapid energy and reduce traveling stress. At all times, food and water were made available. Feeders, waterers, plastic feed buckets, and other tools were thoroughly cleaned, rinsed, and disinfected.

**Table 1:** Ingredient and nutrient composition of self-formulated starter and grower diets (kg/100 kg).

Ingredients	Starter diet (0–14 days)	Grower diet (15–28 d)
Yellow corn	50.93	51.10
Soybean meal	36.00	34.00
Soybean oil	4.000	5.000
Rice polish	4.000	5.000
Limestone	1.400	1.500
Di-calcium phosphate	1.500	1.500
Sodium chloride	0.320	0.350
dl-methionine 99 %	0.500	0.400
l-Lysine 79 %	0.600	0.500
l-threonine 98.5 %	0.400	0.300
Choline chloride 60 %	0.100	0.100
*Vitamin-mineral premix	0.250	0.250
**Nutrient composition (%)**		
Dry matter	88.20	88.30
ME, kcal/kg	3050	3140
Crude protein	22.00	20.40
Fat	09.10	10.00
Crude fiber	03.90	04.00
Ash	04.10	04.60
Calcium	01.10	01.11
Available phosphorus	00.46	00.52
Lysine	00.64	00.59
Methionine	00.55	00.48
Threonine	00.45	00.45
Total	100.0 kg	100.0 kg

ME = Metabolizable energy, kcal = kilo calorie, kg = kilogram; *Broiler premix contained Vitamin A 12.50 MIU, vitamin D 2.50 MIU, vitamin E 25 g vitamin K 4 g, Iron 24 g, Zinc 40 g, Manganese 48 g, Selenium 0.12 g and Cobalt 0.30 g

**Table 2:** Nutrient composition of commercial-ready feed.

Nutrients	Pre-Starter	Starter	Grower
ME (kcal/kg)	2950	3000	3050
Crude protein (%)	21.00	20.00	19.00
Calcium (%)	01.00	00.95	00.95
Phosphorus (%)	00.45	00.45	00.45
Crude Fiber (%)	05.00	05.00	05.00
Lysine (%)	01.15	01.05	01.05
Methionine (%)	00.48	00.45	00.45
Moisture (%)	12.000	12.00	12.00

ME = Metabolizable energy, kcal = kilo calorie, kg = kilogram.

#### 2.2.4 Data collection, processing, and analysis

The field survey data were aggregated, synchronized, and then entered into computers using the MS Excel application (2016). Using an appropriate scoring technique, the qualitative data were converted into quantitative data. For analyzing the data, a combination of descriptive statistics (sums, averages, percentages, etc.) and mathematical techniques were used to obtain meaningful results. For the experimental trial, the body weight (BW), body weight gain (BWG), feed intake (FI), feed conversion ratio (FCR), survivability, and other necessary data of the experimental birds were recorded and calculated weekly, whereas the temperature and relative humidity were recorded daily. Then, all the recorded and calculated data were statistically analyzed using one-way analysis of variance (ANOVA) in SPSS, Statistical Computer Package Program 20.00 (SPSS Inc., Chicago, IL) [15]. Tukey's honestly significant difference test was used to compare variations among the treatments where ANOVA showed significant differences. The level of significance was considered at 95 %.

## 3 Results

### 3.1 Phase-1: farmers’ information and the scenario of using drugs and additives in small- and medium-scale broiler rearing

#### 3.1.1 Farmers’ general information

The survey results clearly showed that the majority of farmers were male, with approximately 82 % of respondents between the ages of 30 and 50. Approximately 65 % of all participants had reached high school (class 6–10), and 46 % owned land ranging from 0.6 to 1.0 acre per family. The majority of farmers (67 %) had farm flocks of 500–1000 birds. According to the current poll, 53 % of farmers used poultry farming as their primary source of income, with approximately 57 % of earnings. We also found that approximately 34 % of farmers had received training from the Youth Training Center, Upazila Livestock Office, or NGOs, while the remaining 66 % had not received such training. Over 88% of broiler farmers rely entirely on local dealers to purchase DOCs, feed, medicines, and other necessary inputs along with a package of advice. Farmers said during face-to-face interviews that the dealers or local agents provided DOCs, feed, medicines, additives, and other inputs as a loan or in exchange for all saleable broilers to them. Under the contract, farmers receive 60 % of the market profit, and dealers receive 40 %. Because profit and loss are inextricably linked, most farmers must endure losses in their businesses as a result of product price fluctuations and excessive feed costs. The contract is not farmer friendly; hence, if the farmers suffer a loss in business, the dealers do not carry any percentage of it, and the farmers must then concede all losses.

#### 3.1.2 Indiscriminate use of drugs and additives in small- and medium-scale broiler farm operation

It has been noted that all the farmers in this survey administer a wide range of drugs, antibiotics, and additives to the birds through drinking water, both at therapeutic and subtherapeutic levels. For the usage of drugs and additives, approximately 62 % of farmers acquired necessary information and consultation from local dealers/agents, another 28 % from experienced neighborhood farmers, and only 10 % sought assistance from a qualified veterinary practitioner. Almost 58 % of farmers used levofloxacin and enrofloxacin, followed by 50 % ciprofloxacin, metronidazole, doxycycline, 48 % amoxicillin, 41 % sulphadiazine (sulfa antibiotics) + trimethoprim, 33 % oxytetracycline and colistin sulfate (Fig. 1). Farmers also used several nonantibiotic drugs and additives. For example, approximately 58 % of farmers used anticoccidial drugs such as amprol, anticocci-k and ervisol, 41 % Tylose MH50 and microtol, 33 % ammo check (anti-ammonia), lysovit (immune stimulator), 25 % brochovet (anti-cold), para plus (paracetamol to reduce cold shock), activate (organic acid), 16 % arolief (anti-respiratory), antox (toxin neutralizer), and TBD (toxin binder) **(**Table 3**)**. In the case of additives or supplements, 100 % of farmers applied glucose-C, followed by 83 % AD_3_E plus, 66 % vitamin-C and thiavin, and 33 % vitamin-K and vita amino **(**Table 3**)**. Furthermore, through on-the-spot observations and interviews, we noticed a routine application of drugs and additives, with most farmers applying the drugs and additives to the broilers even just before or during the time of marketing, without keeping any withdrawal period **(**Table 4**).**

**Table 3:** List of non-antibiotic drugs and additives applied by poultry farmers.

Sl. no.	Generic name of the drug	Drug Description	Percent
1	Amprolium	Anti-coccidial drugs	58.33
2	Sulphadimethoxine, Sulphadimidine, Diaverdine, Nicotinamide, Vitamin K3.	Anticoccidial drug	50.00
3	Tylose MH50-forming laxative	Used to relieve dry, irritated eyes	41.67
4	Mannitol	Toltrazolil	41.67
5	Sodium Benzoate and Sodium Phenylacetate	Anti-ammonia	33.33
6	Ascorbic Acid + Cyanocobalamin + Nicotinamide + Pyridoxine + Riboflavin (Vitamin B2) + Thiamine HCl (Vitamin B1)	Immune stimulator	33.33
7	Ervisol	Anticoccidial drug	25.00
8	Salbutamol	Anti-cold	25.00
9	Paracetamol + Caffeine	Paracetamol	25.00
10	Activate	Organic acids	25.00
11	Arolief	Anti-respiratory drugs	16.67
12	Betacarotene + Vitamin C + Vitamin E.	Toxin nutrilizers	16.67
13	Tetrabenazine	Toxin binder	16.67
**Sl. No.**	**Name of additives or supplements**	**Drug description**	**Percent**
1	Glucose-c	Glucose and Vitamin C	100.0
2	Vitamin C Premix (Ascorbic acid)	Vitamin C	66.67
3	Vitamin-k	Vitamin K	33.33
4	Vita Amino	Vitamin and Amino acids	33.33
5	Thiavin	Vitamin B1 and B2 Premix	66.67
6	AD_3_E plus	Vitamins	83.33
7	Zinc Super- *Pc*	Mineral mixer	50.00

**Table 4:**
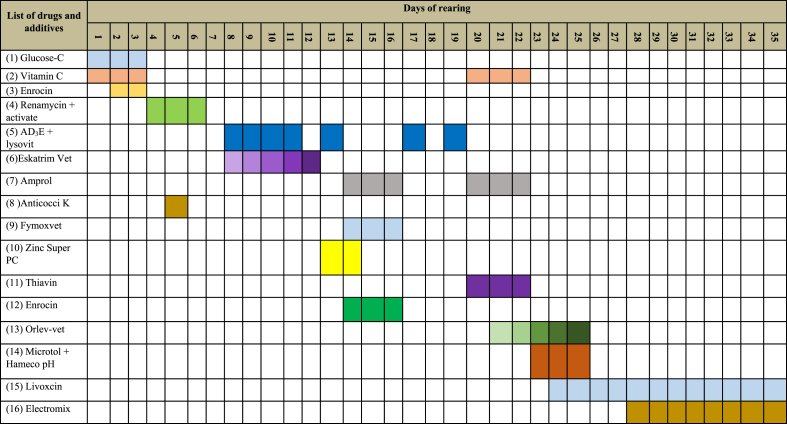
Schedule of using drugs and additives practiced by the small and medium scale farmers in rearing broiler chickens (0–35 days) under rearing areas.

### 3.2 Phase-2: experimental trial for the evaluation of necessity in the use of drugs and additives

#### 3.2.1 Growth performance

##### 3.2.1.1 Body weight

**Fig. 2 (a–d):**
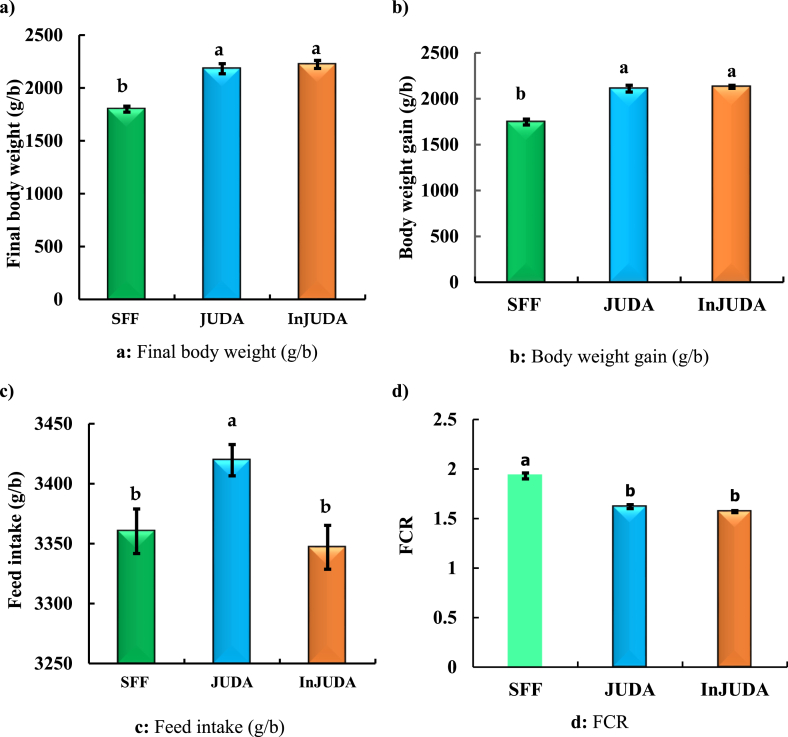
Growth performance of experimental birds, Fig. 2a: Final body weight (g/b), Fig. 2b: Body weight gain (g/b), Fig. 2c: Feed intake (g/b), Fig. 2d: FCR Here, SFF= Self-formulated feed, JUDA = Commercial feed with judicious use of drugs and additives, InJUDA = Commercial feed with injudicious use of drugs and additives, ^a, b^ = means bearing dissimilar superscript differ significantly (p < 0.05).

The results of the experimental trial revealed substantial differences in final body weights among the treatment groups (Fig. 2a). Birds fed SFF attained the lowest final body weight (1799.61 g/bird), followed by the JUDA (2181.94 g/bird) and InJUDA (2222.54 g/bird) groups. The difference in final body weight between the JUDA and InJUDA groups was only 40.6 g/bird.

##### 3.2.1.2 Body weight gain

As with body weight, a similar pattern in final body weight gain was also observed among the treatment groups (Fig. 2b). As expected, the lowest body weight (1746.55 g/bird) was found in the broilers fed SFF, although the differences in body weight gain between JUDA (2110.0 g/bird) and InJUDA (2129.91 g/bird) were minimal and statistically insignificant.

##### 3.2.1.3 Feed intake

The results showed significant (p < 0.05) changes among the treatment groups in terms of feed intake (Fig. 2c). Birds in the JUDA group consumed significantly (p < 0.05) more feed (3419.57 g/bird) than those in the SFF (3360.31 g/bird) and InJUDA groups (3346.90 g/bird).

##### 3.2.1.4 Feed conversion ratio (FCR)

Fig. 2d depicts the feed conversion ratio among the treatment groups. The birds in the SFF group had a significantly (p < 0.05) higher FCR value (1.93) than those in the other treatment groups. The FCR values for the JUDA and InJUDA groups were 1.62 and 1.57, respectively, which is statistically nonsignificant.

#### 3.2.2 Mortality (%)

**Fig. 3:**
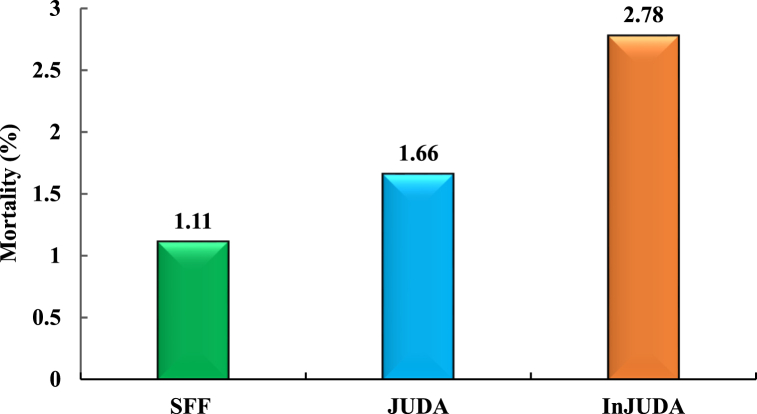
Mortality (%) of experimental birds Here, SFF= Self-formulated feed, JUDA = Commercial feed with judicious use of drugs and additives, InJUDA = Commercial feed with injudicious use of drugs and additives.

The mortality of all treatment groups is displayed in Fig. 3. Birds in the InJUDA group had numerically higher mortality (2.78 %) than those in the JUDA (1.66 %) and SFF groups (1.11 %).

#### 3.2.3 Economic appraisal

Table 5 shows a cost‒benefit analysis of the treatment groups. The feed cost per bird was highest in the JUDA group, followed by the InJUDA group, and lowest in the birds fed SFF. During the experimental period, no medications were used in the SFF or JUDA groups, but in the InJUDA group, indiscriminate use of medicines resulted in a significant (p < 0.05) expenditure of BDT 10.52/bird, which is approximately 5 % of the total production cost. Due to high mortality, the InJUDA group showed significantly (p < 0.05) the highest mortality cost (BDT 7.39/bird) compared to the other treatment groups. The total production cost per bird or per kg live bird was statistically (p < 0.05) higher in the InJUDA group and lowest in the SFF group. The JUDA group obtained statistically the highest profit in terms of both per bird and per kg live bird, whereas the InJUDA group showed significantly (p < 0.05) lowest profit per kg live bird. There is no significant effect on the cost‒benefit ratio among the treatment groups.

**Table 5:** Cost benefit analysis of experimental treatments.

Parameter	SFF (T1)	JUDA (T2)	InJUDA (T3)	*P*-value	Sig.
Feed cost (Tk./bird)	130.51^c^ ± 0.86	160.71^a^ ± 0.03	157.30^b^ ± 0.05	0.02	*
Chick cost (Tk./bird)	22.00	22.00	22.00	–	–
Medicine cost (Tk./bird)	0.00^b^ ± 0.00	0.00^b^ ± 0.00	10.52^a^ ± 0.28	0.01	**
Miscellaneous cost^**(1)**^ (Tk./bird)	15.00	15.00	15.00	–	–
Mortality cost (Tk./bird)	02.38^c^ ± 0.21	04.38^b^ ± 0.34	07.39^a^ ± 0.49	0.03	*
Total cost of production (Tk./bird)	169.89^c^ ± 0.12	202.09^b^ ± 0.04	212.22^a^ ± 0.37	0.01	**
Total cost of production (Tk./kg. body wt.)	94.44^c^ ± 0.28	92.66^b^ ± 0.13	95.51^a^ ± 0.22	0.00	**
Total income (Tk./bird) (Sale price @ BDT 120/kg)	215.88^c^ ± 0.32	261.72^b^ ± 0.44	266.64^a^ ± 0.34	0.01	**
Profit (Tk./bird)	45.99^c^ ± 0.56	59.63^a^ ± 0.21	54.42^b^ ± 0.12	0.01	**
Profit (Tk./kg live bird)	25.56^b^ ± 0.13	27.34^a^ ± 0.32	24.49^c^ ± 0.11	0.01	**
Cost benefit ratio	00.79 ± 0.01	00.77 ± 0.01	00.80 ± 0.02	0.29	NS

SFF= Self-formulated feed, JUDA = Commercial feed with judicious use of drugs and additives, InJUDA = Commercial feed with injudicious use of drugs and additives;^**(1)**^ vaccines, disinfectants, transport, bedding materials, labor etc.; BDT=Bangladeshi taka; a, b, c = means bearing dissimilar superscript differ significantly (p < 0.05). (±) indicates standard error of mean (SEM).

## 4 Discussions

### 4.1 Phase-1: present scenario on the use of drugs and additives in small- and medium-scale poultry farms

Survey results on the small- and medium-scale poultry farmers demonstrated that a major portion of the farmers had little education with almost no training in poultry farming. According to Imam et al. [16], the majority of farmers do not adhere to the regulations set forth by the Bangladesh Department of Livestock Services and do not sufficiently conduct biosecurity measures. The results of this study showed that female farmers were more likely to use antibiotics inappropriately than male farmers. This may be related to female farmers' inadequate understanding of antimicrobial use and antimicrobial resistance, which is consistent with the reports of other findings [[17], [18], [19]]. However, the farmers who are involved in farming for a fairly long period have good experiences in overall bird management, rearing, medication, or marketing live birds. The majority of marginal farmers are poor, and they lack the financing to purchase basic inputs such as feed, chicks, medications, additives, and so on to run their businesses smoothly. As a result, they must rely entirely on dealers/other local agents for the purchase of day-old chicks (DOCs), feeds, needed treatments and medicines, and even the marketing of live birds. Masud et al. [3] classified such a relationship between farmers and dealers as a 'client-patron’ relationship, in which approximately 95 % of farmers have no choice of selecting quality chicks, quality feeds, or any other inputs; rather, they must acquire whatever the dealer offers [5]. Masud et al. [3] also described that approximately 80 % of poultry farmers learn how to maintain their flocks from dealers and representatives, whereas only 20 % of farmers consult with a veterinarian if they are unable to prevent sickness and chicken deaths. In total, 30 % of dealers provide farmers with the prescription-free medications they want. Approximately 50 % of feed and chick vendors said they do not provide medical care, while the other 50 % were involved in primary care and offered medication depending on their expertise. Eighty percent of dealers and representatives claimed that farmers did complain about the failure of treatment. In that situation, the majority of the sellers (40 %) advised another medication. Most of the vendors who sold antibiotics gave the farmers advice on finishing the course of medication. Again, according to Tasmim et al. [20], more than half of the participants (57.1 %) accepted dealer advice to give their birds antibiotics, and 54.2 % of farmers purchased such drugs directly from the dealers. However, only 15.7 % and 10.0 % of farmers purchased antibiotics as recommended by the government and private veterinary professionals, respectively. Furthermore, during the course of this poll, 97.1 % of farmers recognized that such overuse of antibiotics in chickens is harmful to humans. According to the findings of the current survey, the majority of poultry feed and chick dealers have no professional degrees in the veterinary sector, but they recommend antibiotics to farmers based on their experience (usually 3–10 years). One of the main issues that could encourage the overuse of antibiotics in veterinary and human medicine in Bangladesh and several other developing nations is the over-the-counter sale of antibiotics [3,[21], [22], [23]]. In the current survey, we also found that poor farmers were obtruded by dealers/local agents to sell their live birds to them, as the farmers received DOCs, feed, medicines, and other necessary inputs as loans. Despite such unpaid credit, dealers/local agents imbibed a certain percentage of profit from farmers with such an unfair contact, where the dealers never incurred any loss, but the farmers must have endured all kinds of risk for each batch of broilers. Similar to this statement, Masud et al. [3] clearly stated that farmers purchased DOCs, feed, drugs, additives, and other inputs on credit from dealers; thus, they have no opinion or option to choose the quality of the product, and finally, they must repay the credit by selling ready birds to the dealers. Furthermore, past research indicates that approximately 92 % of farmers rely on local agents/middlemen to market their birds, with 88 % of farmers failing to make the desired profit [5]. As a result, it is clear that the small and medium-scale poultry farmers in Bangladesh are being exploited by dealers/agents, which is linked to the inputs-outputs marketing system, and farmers eventually had to concede a loss in their business. It should be highlighted that only a small number of farmers may be able to acquire the aforementioned inputs entirely or partially in cash.

Corroborating the present survey report, Masud et al. [3] further mentioned that more than 50 % of farmers applied levofloxacin, ciprofloxacin, metronidazole, enrofloxacin, and doxycycline without consultation to a registered veterinary practitioner. Supporting the present survey result, Rousham et al. [24] also stated that antibiotics were used by 95 % of broiler farms, and 80 % of those farms used several antibiotics. The most common medication given was tetracycline (63 %), followed by ciproﬂoxacin (55 %), enroﬂoxacin (55 %), erythromycin (38 %), tylosin (38 %), and colistin sulfate (15 %).

In general, dealers or medicine representatives have a monthly or yearly selling target, so they tempted the farmers to use various drugs and additives indiscriminately to meet their sales target or even suggest that the farmers use drugs and additives without regard for the withdrawal period for a specific antibiotic (Table 4). Similar to this research, Masud et al. [3] found that farmers began administering antibiotics on day one and continued until the birds were sold, based on the dealer's recommendation. In almost all cases, farmers had no idea why they were using such drugs and additives, but they deceptively believed that utilizing such drugs and additives on a regular basis for the full duration of rearing resulted in better growth and ensured birds' safety from disease outbreaks. Tasmim et al. [20] previously indicated that commercial poultry producers (54 %) started using antibiotics on the first day of the chicken production cycle, not only as a prophylactic measure (43 %) but also as a growth enhancer (4 %). In a recent report, Imam et al. [16] suggested that almost 100 % of broiler and layer farms used antibiotics in the current production cycle. Moreover, 18 % of the broiler farmers and 13 % of the layer farmers used antibiotics for prophylactic purposes. Regarding the use of antibiotics by small- and medium-scale farmers, Tasmim et al. [20] stated that approximately 98.6 % of farmers were unaware of antimicrobial resistance (AMR), 47.1 % of farmers were unable to explain antibiotics, 42.9 % used antibiotics for preventative purposes, 4.3 % used them to promote growth, 25.7 % used antibiotics recommended by veterinarians, 42.9 % used leftover antibiotics, and 50 % did not maintain the antibiotic residual withdrawal period. In general, farmers in underdeveloped and/or developing countries use antibiotics to enhance growth without consulting registered veterinarians. Surprisingly, over 88 % of the poultry producers did not follow the needed antibiotic withdrawal period before marketing the birds [25,26]. In a different study, Sultan et al. [27] found that only 10 % of farmers stopped using antibiotics prior to marketing the birds, and only 2 % withdrew antibiotics at least seven days before selling, despite failing to follow the mandatory standard withdrawal schedule. Moreover, antibiotic resistance may develop in Bangladesh as a result of indiscriminate use of antibiotics by unqualified providers, irrational antibiotic distribution by animal feed dealers and drug sellers, aggressive marketing, inadequate information on the antibiotic use guidelines, and ineffective regulation implementation [28]. Various published research reports clearly demonstrated widespread availability and unjustifiable use of antimicrobials in poultry, large animals, and even humans in the country without a prescription, which is undoubtedly a crucial fact of deep-rooted supply- and demand-side factors. To address these issues, we have recommended that the relevant authorities of the Government of the People's Republic of Bangladesh develop an 'action plan’ to rebuild public awareness, utilize adequate resources, and implement national surveillance to reduce the inappropriate and unnecessary use of antimicrobials.

### 4.2 Phase-2: experimental trial for necessity evaluation on the use of listed drugs and additives in small-scale broiler production

Based on our survey results and other scientific reports, it is clear that the small-scale broiler farmers in Bangladesh utilized a variety of medications and additives injudiciously throughout the entire rearing period under the influence of dealers or medical representatives. In the second phase, we ran a feeding trial at University Poultry Farm to determine whether the inclusion of such pharmaceuticals and additives is indeed necessary for the birds or if the farmers simply utilized the drugs injudiciously after being persuaded by the dealers. The results clearly demonstrated that there were no significant differences between birds fed commercial feed with judicious (JUDA) and injudicious (InJUDA) use of drugs and additives, suggesting that the drugs and additives used by small-scale farmers in broiler production are most likely unnecessary; they were simply exploited by local dealers in the usage of such abundant drugs and additives through drinking water that accounts for almost 6–8% of the total production cost, the majority of which could be added to the usual profit margin of their business. Miles et al. [29] found that supplementing two antibiotics, bacitracin and virginiamycin, in male broilers as a growth promoter resulted in only 47 g and 33 g greater body weights than the control at 7 weeks of age. The addition of 0.1 % flavomycin to the basal diet showed no effect on broiler body weight, weight gain, feed consumption, or FCR [30]. Baurhoo et al. [31] also found no significant impact of antibiotic growth promoters on the body weight and FCR of broilers. However, the administration of antibiotics such as penicillin, oxytetracycline, lincomycin, bambermycin, and tylan as growth promoters resulted in a small improvement in the body weight of broilers (10–18 %) [32]. When compared to conventional rearing, birds kept complete drug-free management lost just 50 g (2.06 %) of body weight [33]. Furthermore, published studies revealed that it is possible to economically rear broiler chickens utilizing a drug-free approach; however, this appears to be associated with lower performance [34,35]. In the present study, when the birds were reared almost drug-free, the total production performance of commercial broiler chickens was likewise marginally reduced (Fig. 2a). Thus, the results of previous studies corroborate the findings of our current experiment that indiscriminate supplementation with drugs and additives, particularly antibiotics, has very little or no impact on the growth performance of birds. It is unclear why the injudicious and abundant use of drugs (specifically antibiotics) in the current study had no significant impact on broiler growth; however, it has been suggested that long-term use of antibiotics may lead to disrupted beneficial bacteria [36,37], multiple catabolic disorders and immunosuppressive effects [12], which all together may negatively affect host health, rendering the birds highly vulnerable to diseases, leading to poor nutrient digestion, absorption and utilization.

Furthermore, the experimental birds fed SFF showed the expectedly lowest growth performance compared to the other two treatment groups. Poor digestibility, improper mixing of feed ingredients, selective feeding, differences in feed forms (mash, crumble, or pellet), and other factors could all contribute to the lower growth performance observed in the SFF-fed birds. Additionally, it is well known that feed millers include a variety of supplements in the formulation of commercial feeds, which might also contribute to the better growth outcomes seen in commercial feed-based JUDA and InJUDA diets.

It remains unclear why a greater number of birds died after injudicious drug and additive supplementation; however, it is possible that the indiscriminate use of medicines made the birds physiologically vulnerable and thus unable to cope with the handling, catching, or other stresses that evolved, particularly at the later stage of their life, resulting in sudden death. Almost similar results were postulated by Gunal et al. [30], who found higher mortality after supplementation with an antibiotic growth promoter (flavomycin) compared to the control. According to our practical and visual observations, the birds fed SFF or supplemented with JUDA were more attentive and active and had clean and shiny plumage. Notably, mortality in these two groups occurred only during the initial weeks of rearing. The indiscriminate use of medications and additives in the InJUDA group incurred approximately 5 % of the overall production cost, which when combined with the mortality loss resulted in significantly higher production costs (BDT 95.51/-) and the lowest profit (BDT 24.49/-) per kg of birds. In contrast, significantly, the highest profit was achieved in the JUDA group of birds, in which drugs and additives were used sparingly as prescribed by a registered veterinarian. Antibiotics are largely employed in the chicken diet at subtherapeutic levels to address subclinical infections and preserve the intestinal health of the birds [38] and are thus typically advised to be used judiciously to prevent or cure any disease occurrence. However, excessive and injudicious use of drugs and additives, including antibiotics, does not improve the growth of birds, as expected, but rather affects the total cost of production. Likewise, although the irrational use of drugs and additives very slightly improves the growth of broilers, considering mortality loss and cost incurred for drugs and additives, such a small enhancement does not yield a satisfactory profit over the cost. The use of antimicrobials is only profitable up to a certain threshold because there is an inverted U-shaped relationship between antimicrobial use and financial outcomes [39,40]. Therefore, the injudicious use of drugs, additives, or other chemicals in small-scale farm operations is not only meaningless but also wastes a large amount of money that could otherwise be added to the farmer's profit margin. In addition, such malpractice in broiler production may reduce overall meat quality, create microbial dysbiosis in the gut, and lead to antimicrobial resistance, all of which may have a detrimental impact on consumer attitudes toward meat and meat products. The aim of the current study was therefore to evaluate how much economic value was obtained as a result of the excess gain of broilers due to the injudicious and abundant use of drugs and additives, which has yet to be justified in any previous studies.

Presently, Bangladesh produces approximately 17.5–20.0 million broiler DOCs every week. Based on a rough calculation, we estimated a large amount of annual loss due to the unnecessary use of drugs and additives, which must eventually be borne by small- and medium-scale broiler farmers. In agreement with the results of our current study, Graham et al. [41] claimed that positive production changes with the use of antibiotic growth promoters were incommensurate to compensate for the cost of antibiotics and declared that the antibiotic growth promoter alone was forfeited approximately 0.45 % of the total cost per chicken. Based on our experience, superior husbandry practices, with a focus on brooding, biosecurity, litter management, lighting, and feeding of the experimental birds, may result in expected production performance and lower mortality. However, it is ironic that small-scale farmers vehemently refused to believe that profitable broiler farming could be achieved only with sound husbandry practices and avoiding the indiscriminate use of drugs and additives. Thus, rebuilding farmers' trust in fair farm practices is the key challenge of today's broiler farm operation.

## 5 Conclusions

In conclusion, small- and medium-scale farmers are urged to avoid the injudicious use of drugs and additives in farm operations for sustainable, profitable, safe, quality broiler production and should rely on standard husbandry practices and biosecurity. Furthermore, small-scale farmers should not completely rely on dealers/agents under the so-called 'client-patron’ relationship, in which they are constantly exploited in various ways by dealers or agents who have filched a large sum of money. Farmers should only use medications and additives after consulting with an authorized veterinary practitioner.

## Funding statement

This research was funded by 10.13039/100019278Bangladesh Agricultural University Research System (BAURES) within the framework of the research project “Quality and safety assurance of broiler meat production under small and medium scale farming in Bangladesh” (Project ID: 2018/627/BAU).

## Data availability statement

Data included in this article are available to the corresponding author and can be produced on request.

## Additional information

No additional information is available for this paper.

## CRediT authorship contribution statement

**Shubash Chandra Das:** Writing – review & editing, Writing – original draft, Supervision, Conceptualization. **Mosa Zubiatin Tasmin:** Visualization, Data curation. **Afifa Afrin:** Writing – original draft, Visualization, Methodology, Data curation. **Tanvir Ahmed:** Writing – review & editing, Writing – original draft, Formal analysis, Data curation. **Ankon Lahiry:** Writing – review & editing, Writing – original draft, Data curation. **Shahina Rahman:** Writing – review & editing.

## Declaration of competing interest

The authors declare that they have no known competing financial interests or personal relationships that could have appeared to influence the work reported in this paper.
