# Supplementary material for: Challenges in the profitability of small-scale broiler farming by avoiding injudicious use of drugs and additives
Source: Heliyon. 2024 Jan 23;10(3):e25001. doi: 10.1016/j.heliyon.2024.e25001 (PMC10839958; doi:10.1016/j.heliyon.2024.e25001)
Supplement: Multimedia component 1 [file mmc1.pdf]

QUESTIONNAIRE

DEPARTMENT OF POULTRY SCIENCE

BANGLADESH AGRICULTURAL UNIVERSITY, MYMENSINGH

Questionnaire (English Version) for conducting survey to assay status of commercial broiler farming at different location of Mymensingh and Jamalpur division.

Chapter 1: General Information

1. Name :

2. Husband/Father name:

3. Address

Village: Thana: Post office: District :

4. Educational background:

(a) Illiterate (b) Primary Eductaion (c) Secondary Eductaion (d)Higher Secondary Education (e) Graduation (f) Others.....................................

Chapter 2: Family Demographic Information

SL. No.	Name	Age	Sex	Relation with the family Head	Educational Background	Marital Status	Occupation	Voter
1								
2								
3								
4								
5								
6								
7								
8								
9								
10								

Occupational code 1 = Housewife, 2 = Landless farmer, 3 = Agricultural daily wage laborers, 4=Non-Agricultural daily wage laborers, 5 = Farm business, 6 = Chicken/Duck rearing, 7= Cow/goat rearing, 8 = Small business, 9 = Student, 10= Older person, 11= Unemployed, 12= Others

Chapter 3: Details about animal resources

Breed	Deshi Breed		Hybrid		Ownership Male= 1 Female=2 Common=3	Sex of the rearer Male= 1 Female=2 Common=3	Own=1 Lease=2
	Numer	Amount (Taka)	Numer	Amount (Taka)			
Drake							
Duck							
Cockerel(Male under 12 months)							
Pullet(Female under 12 months)							
Ducklings							
Geese							
Cow							
Bull							
Bullock							
Milking cow							
Heifer cow							
Buck (Male goat)							
Doe(Female goat)							
Wether (castrated goat)							
Kid							
Others							
Sheep							
Buffalo							
Cock							
Hen							
Others							

Chapter 4: Family Income Information

Source of income	Amount (Taka)	Others
Agricultural daily wage laborers		
Non-Agricultural daily wage laborers		
Chicken/Duck rearing		
Cow/goat rearing		
Business		
Others		
Total income		

Chapter 5: Information on broiler farming

1. How long ago did you start a broiler farm?

....

2. Have you received any training in broiler farming?

(a) Yes (b) No

3. Where did you take training from?

(a) Upazila Livestock Office (b) NGO (c)Youth Development Organization

(d) Others ....

4. What was the duration of training?

(a) 7 days (b) 15 days (c) 1 month (d) 3 months (e) Others.............................

5. How much did you spend on shed preparation?

....

6. What other things cost you except shed?

....

7. What is the size of the shed?

....

8. How many broilers do you usually rear per batch?

(a) 500 (b) 1000 (c) 1500 (d)2000 (e) Others...................................

9. Which strain of broiler do you rear?

(a) Cobb 500 (b) Hubbard classic (c) Indian river meat (d) Others...................................

10. What is the price of chick?

....

11. Who is the supplier of broiler DOCs?

(a) Veterinary Surgeon (b) Dealer (c) Village quack (d) Others..................

12. How much feed do you provide (g/bird/day)?

....

13. What is the price of feed per kg?

....

14. Who is the supplier of broiler feed?

(a) Veterinary Surgeon (b) Dealer (c) Village quack (d) Others..................

15. What is the input (DOC, feed and medicine) purchasing criteria for broiler production?

(a) On cash (b) As loan (c) Other dealing......

16. From whom do you take suggestions when there is a disease on the farm?

(a) Veterinary Surgeon (b) Dealer (c) Village quack (d)Others...................................

17. Do you have to pay any fees to doctors after visiting your farm?

(a) Yes (b)No

18. What is the interval period you usually follow between two batches of broiler production?

(a) 5 days (b) 10 days (c) 14 days (d) 20 days (e)Others...................................

19. What components do you use for cleaning your broiler shed?

....

20. Do you use a footbath on the farm?

(a) Yes (b)No

21. Do you spray any disinfectant around the farm?

(a) Yes (b)No

22. Do you control the temperature of the broiler shed?

(a) Yes (b)No

23. What materials do you use as litter?

(a) Rice husk (b) Saw dust (c) Sand (d) Others

24. Do you use the same litter more than once?

(a) Yes (b)No

25. Do you perform racking of litter ?

....

26. What is the source of water on the farm?

....

27. What vaccination schedule do you follow for broilers from day one to market age?

Day	Name of the vaccine	Name of the company	Dose	Price
1				
2				
3				
4				
5				
6				
7				
8				
9				
10				
11				
12				
13				
14				
15				
16				

17				
18				
19				
20				
21				
22				
23				
24				
25				
26				
27				
28				
29				
30				
31				
32				
33				
34				
35				

28. What medicines do you supply to broilers from day one to market age?

Day	Name of the vaccine	Name of the company	Dose	Price
1				
2				
3				
4				
5				
6				
7				
8				
9				
10				
11				
12				
13				
14				
15				
16				
17				
18				
19				
20				

21				
22				
23				
24				
25				
26				
27				
28				
29				
30				
31				
32				
33				
34				
35				

29. Who prescribed you to use these vaccines and medicines during broiler farming?

(a) Dealer (b) Medical representative (c) Veterinary doctor (d) Others..........
